# Cervical vagal schwannoma, a rare differential of a neck swelling: A case report

**DOI:** 10.1002/ccr3.6560

**Published:** 2022-11-12

**Authors:** Ramesh Prajapati, Aakar Thapa, Jayant Kumar Yadav, Manik Lama, Prakash Shrestha, Ashmita KC, Pritam Gurung, Basant Pant

**Affiliations:** ^1^ Annapurna Neurological Institute and Allied Sciences Kathmandu Nepal; ^2^ Tribhuvan University, Institute of Medicine Kathmandu Nepal; ^3^ National Center for Rheumatological Disease Kathmandu Nepal

**Keywords:** cervical schwannoma, vagal schwannoma

## Abstract

Cervical vagal schwannoma is a rare clinical entity that requires a different clinical approach than other neck swellings. Magnetic resonance imaging is the preferred initial diagnostic test. Complications may arise due to vagal stimulation in unsuspecting open biopsies. Surgical excision with perioperative vagal monitoring is recommended for the treatment of vagal schwannomas.

## Graphical abstract

Cervical vagal schwannoma is a rare differential of neck swelling. The usual approach to neck swelling is ultrasonography and, if required, FNAC for diagnosis. However, biopsy should be avoided in cases of cervical vagal schwannoma.

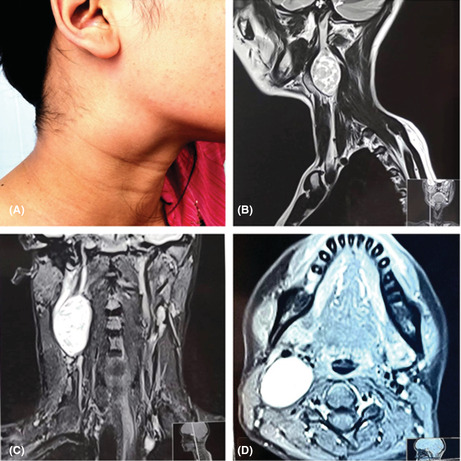

## 1 BACKGROUND

Schwannomas are well encapsulated, mostly benign neurogenic tumors that can arise from cranial, peripheral, or autonomic nerves.^1^ Cervical vagal schwannoma is a rare clinical entity and requires a different approach from other neck swellings. Ultrasonography and fine needle aspiration (FNA) are initial diagnostic means in the evaluation of neck lumps. However, they do not provide an effective preoperative diagnosis of vagal schwannomas. Further, cardiac arrests due to vagal stimulation have been reported in unsuspecting open biopsies. Therefore, Magnetic resonance imaging (MRI) is the preferred initial diagnostic test.

We herein report a case of a young female patient who presented with progressive neck swelling and was diagnosed with vagal schwannoma. We further reviewed the literature and described the approach to the diagnosis, surgical management, and possible operative complications of a vagal schwannoma.

## 2 CASE PRESENTATION

A 24‐year‐old lady presented to our institute with a 3‐year history of painless and progressive swelling in her right neck. She did not have difficulty breathing or swallowing and had no change in her voice. She had no change in her body weight and appetite, no cough, night sweats, or on/off fever. She had no prior medical history of significance and had no previous exposure to tuberculosis (TB). There was no history of similar swelling or any neurogenic tumors in the patient's family.

The lump was noticeable on the right level 2 region of the neck and felt firm on touch, was non‐tender, mobile on a horizontal plane, well‐demarcated, and measured about 4 × 3 cm in dimensions. There was no cough on palpation, and the swelling did not move with deglutition. Oral examination revealed no distortion of the anatomy. Ear examination and hearing were regular. No other findings were noted on systemic examination.

**FIGURE 1:**
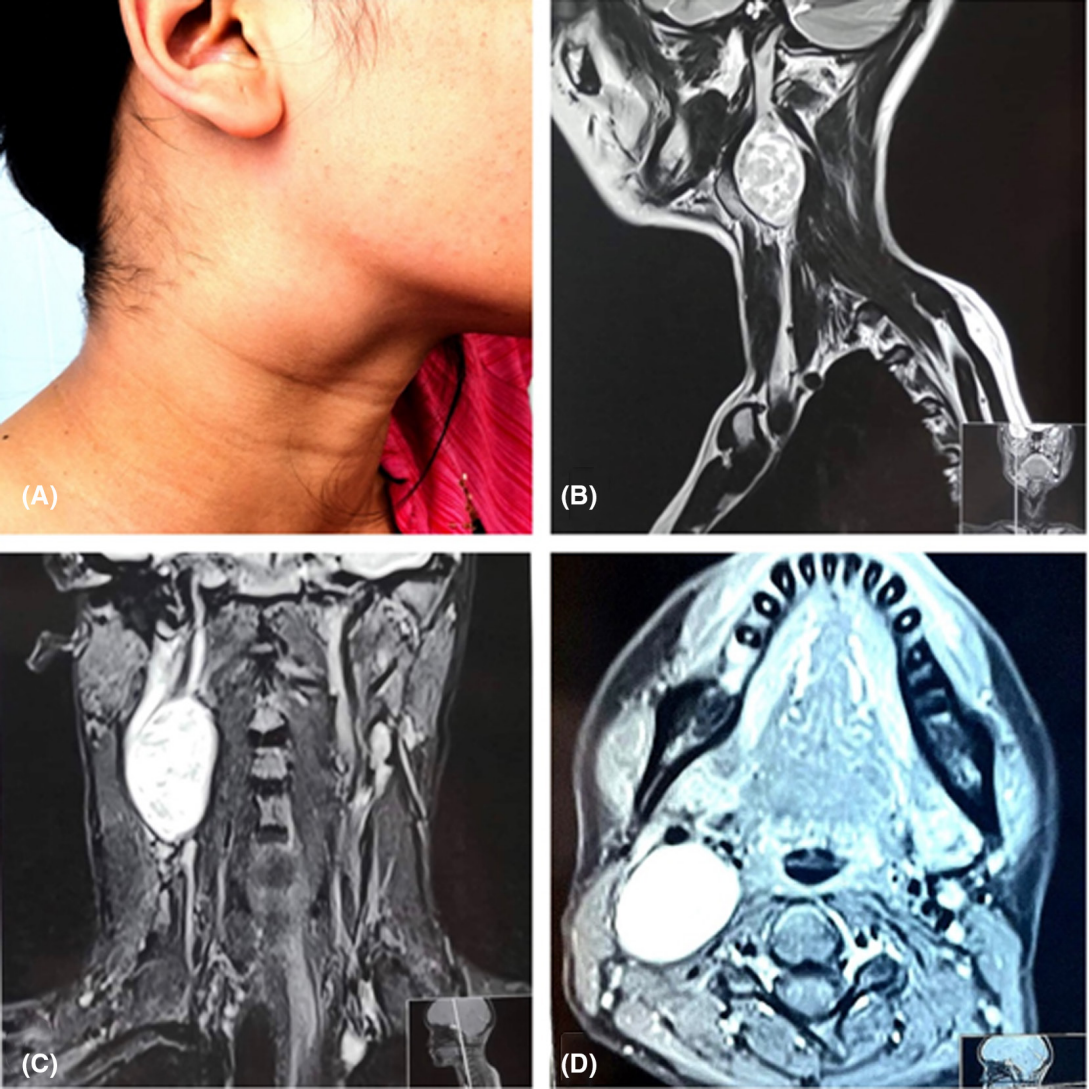
(A) Swelling noted in neck, (B–D) MRI T2 Sagittal, coronal, and axial section respectively showing well defined lobulated lesion (2.4 × 2.4 × 2.8 cm), heterogeneous signal changes in right lateral neck in carotid space. The lesion is displacing the internal carotid artery and internal jugular vein anteriorly without luminal narrowing. Laterally the lesion is abutting sternocleidomastoid muscle with maintained fat plane.

With the suspicion that the swelling arose from a level 2 lymph node, an ultrasonography‐guided fine needle aspiration biopsy (FNAB) was arranged. The tissue sample was sent for histopathological examination, TB smear, and culture for TB and GeneXpert. The reports came in negative for TB, and the biopsy reports were consistent with Schwannoma with tumor cells staining positive for IH558 and S‐100. Other routine blood investigations and chest X‐rays showed no findings. An MRI of the neck was then arranged to find out the extent of swelling, which revealed a 2.4 × 2.4 × 2.8 cm sized T1 iso, T2/STIR heterogeneously high oblong shaped lesion displacing the internal carotid artery, external carotid artery and internal jugular vein anteriorly, and avid heterogeneous enhancing lesion in right carotid space suggestive of vagal schwannoma (Figure 1). With the FNAB and MRI findings, a diagnosis of vagal schwannoma was made and planned for excision of the tumor.

**FIGURE 2:**
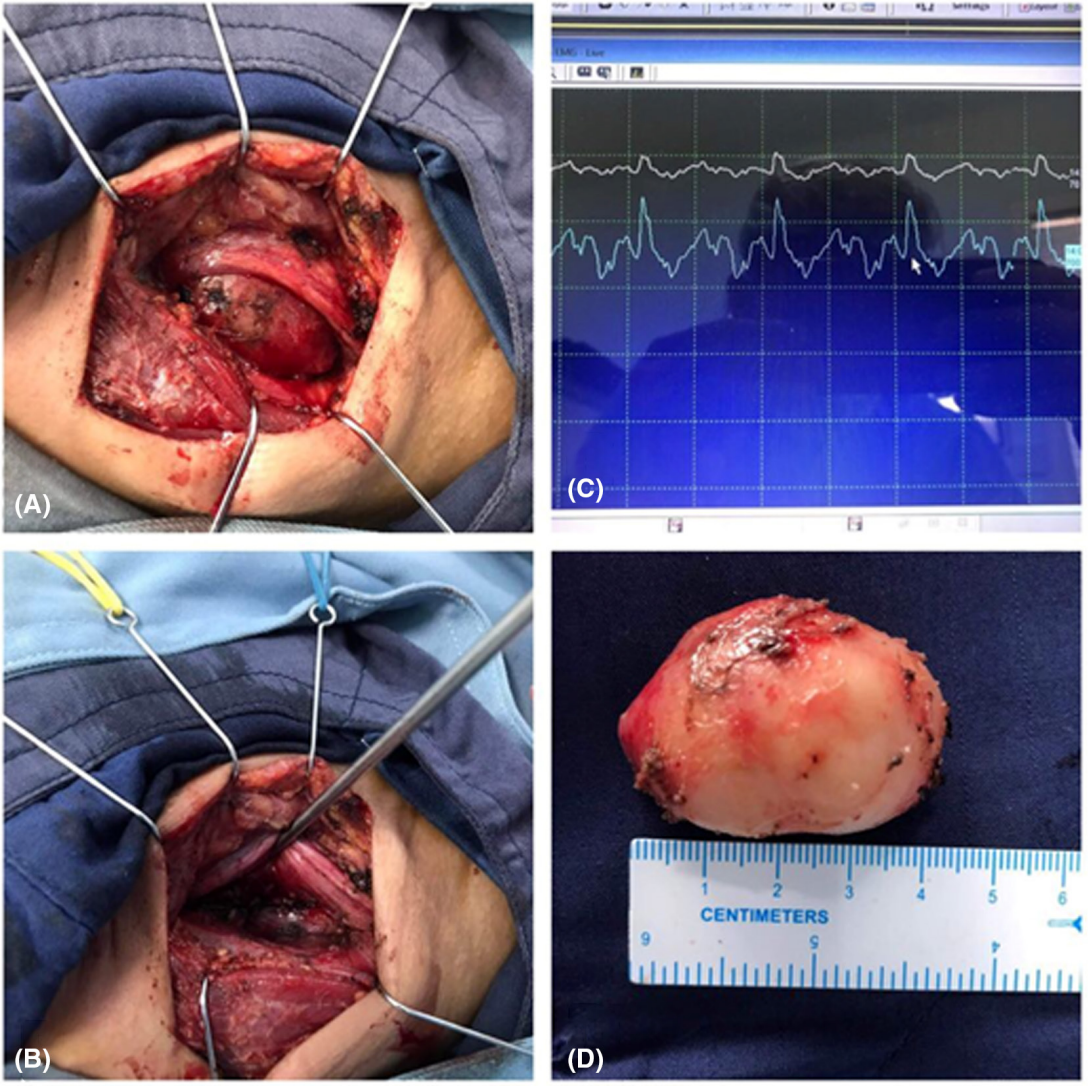
(A, B) Intraoperative view (C) Continuous monitoring of Vagal nerve (D) Gross specimen of vagal schwannoma.

**FIGURE 3:**
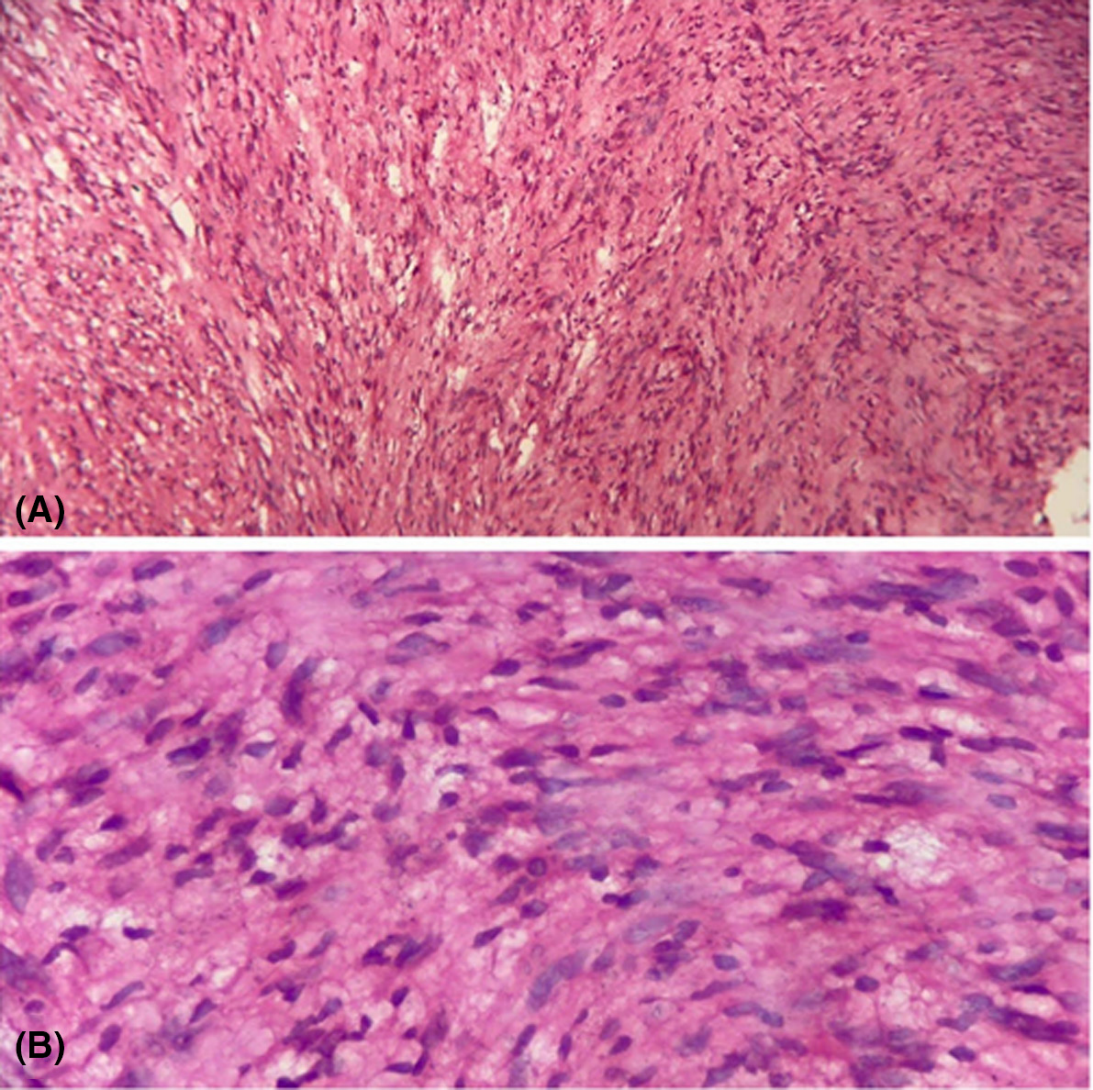
Histopathological examination showing encapsulated tissue and the cells are arranged in diffuse pattern. There are cellular and hypocellular areas with spindle cells and elongated nuclei in a fibrillary background. Other area shows hypocellular area with lymphocytes and lipidized cells. Cellular areas show some of the cells are mild pleomorphic. Other area shows nuclear palisading, ecstatic and hyalinized vessels. (A) Magnification 10×; (B) Magnification 40×.

Intraoperatively, the mass was attached to the nerve sheath, posterior to the carotid artery. Therefore, careful dissection of swelling was done due to its proximity to major vessels and nerves. Vagal monitoring was done throughout the procedure to avoid any autonomic complications, and minimize nerve injury, primarily to reduce postop complications like hoarseness. Approximately 4 × 3 × 2 cm mass, whitish in color, soft to firm non‐vascular was excised (Figure 2). The excised mass was sent for histopathological examination, revealing WHO grade I schwannoma (Figure 3). She was discharged with good postoperative outcomes with no hoarseness. Follow‐up a week later revealed no complications.

## 3 DISCUSSION

Schwannomas are well encapsulated, mostly benign neurogenic tumors that share their origin from nerve sheath cells with neurofibroma. They can arise from cranial, peripheral, or autonomic nerves.^1^ Schwannomas arising in the head and neck region are rare, and rarer is their origin from the cervical vagus nerve. It commonly occurs in the third to fifth decades of life with no gender predisposition.^2^, ^3^ Most cervical vagal schwannomas present as a slowly growing painless lump in the neck with mobility only along the horizontal axis. Cough on palpation is an occasional yet pathognomonic finding. Other presentations could be dysphonia, dysphagia, tongue weakness, and Horner's syndrome depending on the size and extent of swelling.^4^ Pain and nerve deficits point toward malignancy which is associated with neurofibromatosis.^5^, ^6^ The diagnosis can be difficult due to broad differential diagnosis and nonspecific clinical presentation. The differentials to consider are branchial cleft cyst, vascular malformations, reactive lymphadenopathy, lymphoma, carotid artery aneurysm, salivary gland tumors, neurofibroma, metastatic lymph nodes, schwannomas of cervical sympathetic chain, and TB lymphadenitis.^1^, ^7^, ^8^

The paucity of differentiating features can pose a diagnostic challenge. Still, preoperative diagnosis is essential to plan the surgery accordingly and to counsel the patient regarding the possible risk of vocal card palsy.^5^ Ultrasonography and Fine needle aspiration (FNA) are one of the initial diagnostic means for the evaluation of neck lumps. However, they do not provide an effective preoperative diagnosis of vagal schwannomas.^9^, ^10^ Open biopsy is not recommended because of the scarring and loss of surgical plane.^1^ Further, there have been reports of cardiac arrest due to vagal stimulation in unsuspecting open biopsies.^11^

Tubercular lymphadenitis is one of the most common differentials of neck swelling in endemic regions of South Asia. Therefore, we proceeded with needle biopsy with the view to rule out lymph node pathologies such as TB lymphadenitis, lymphoma, and metastases. However, the histopathological findings and immunohistochemistry findings suggested schwannoma. Areas of high cellularity (Antoni A) interspersed with areas of sparse cells (Antoni B) are classical microscopic pattern**s** observed in schwannomas. These also demonstrate strong positivity for S100 staining.^7^ MRI is the investigation of choice for the diagnosis of neurogenic vagal tumors. MRI findings include isointense T1 signals relative to skeletal muscle and increased and slightly heterogeneous T2 signals.^12^ MRI is also essential for suspecting surgical complications and ruling differentials out. Splaying the internal jugular vein and internal carotid artery by schwannoma helps differentiate schwannomas of vagal origin from that of the cervical sympathetic chain.^13^ The distinction from paragangliomas is essential and can be made by intense enhancement on CT and a characteristic “salt and pepper” appearance on MR images of paragangliomas.^14^ It helps in averting hypertensive crisis, which could be encountered if FNA or surgery is performed without due consideration of a neck mass, which turns out to be a hyper‐functional paraganglioma.^9^

The treatment of choice for cervical vagal schwannoma is intracapsular enucleation with nerve‐sparing techniques. End‐to‐end anastomosis or nerve grafting may be mandated in special cases where nerve preservation is not possible.^3^, ^5^ Although wait and watch could be an option for benign, slow‐growing asymptomatic tumors, they eventually encroach upon surrounding structures, and thus surgery is preferred, particularly in the younger age group.^11^ Postoperatively, hoarseness is the most common symptom and can occur with or without coughing, choking, or Horner's syndrome.^8^ Meticulous microsurgical dissection in conjunction with intraoperative nerve monitoring produces a better outcome in terms of postoperative morbidity.^15^

## 4 CONCLUSIONS

Vagal schwannoma is a rare clinical entity that should be considered a differential for slow‐growing neck lump, mainly when arising anterior to the sternocleidomastoid muscle and mobile in a single horizontal plane. MRI is the preferred diagnostic modality that also helps rule out mimics such as paraganglioma and sympathetic chain schwannoma. It also informs the surgeon regarding postoperative vocal palsy. FNA and biopsy are best avoided even though they are frequently performed in evaluating other neck masses. Intracapsular enucleation with intraoperative nerve monitoring provides a better outcome.

## AUTHOR CONTRIBUTIONS

RP, AT, JKY, and AK wrote the draft manuscript. ML, PS, and PG revised the manuscript. All except AT and AK were involved in the patient's direct care. All authors agreed on the final draft for submission.

## CONFLICT OF INTEREST

None of the authors has any conflict of interest to disclose.

## ETHICS STATEMENT

Ethics approval of a case report is not needed in accordance with local ethical guidelines.

## CONSENT

The patient provided written informed consent for publication of this case report and accompanying images.

## Data Availability

Not applicable.
